# Maternal substance use during pregnancy and associated factors in Adama, central Ethiopia

**DOI:** 10.3389/fgwh.2025.1540814

**Published:** 2025-02-27

**Authors:** Kimiya Mohammed, Mihiret Shawel Getahun, Yohanes Abera Belachwe, Nesra Mohammed Fati, Yohannes Mekuria Negussie

**Affiliations:** ^1^ Department of Nursing, Adama General Hospital and Medical College, Adama, Ethiopia; ^2^ Department of Public Health, Adama General Hospital and Medical College, Adama, Ethiopia; ^3^ Department of Biomedical Sciences, Adama Hospital Medical College, Adama, Ethiopia; ^4^ Department of Medicine, Adama General Hospital and Medical College, Adama, Ethiopia

**Keywords:** Ethiopia, maternal substance use, pregnancy, public health, Adama

## Abstract

**Background:**

Substance use, including tobacco, alcohol, khat, and illicit drugs, is a significant preventable contributor to the global burden of disease. Women are particularly vulnerable during their reproductive years, with substance use during pregnancy posing serious risks to maternal and fetal health. Therefore, this study aimed to assess of prevalence of substance use and determine associated risk factors among pregnant women in Adama, Central Ethiopia.

**Methods:**

A health facility-based cross-sectional study was conducted among 472 pregnant women. Participants were selected using a systematic sampling technique. Data were collected through an interviewer-administered structured questionnaire. Binary logistic regression was employed to model the association between substance use and explanatory variables. Adjusted odds ratios (AORs) with 95% confidence intervals (CIs) were used to estimate the strength of association. Statistical significance was determined at a *p*-value < 0.05.

**Result:**

The overall prevalence of substance use during pregnancy was 22.0% (95% CI: 18.2–26.0). Unplanned pregnancy (AOR = 3.49, 95% CI: 1.23–9.89), pre-pregnancy substance use (AOR = 24.16, 95% CI: 11.49–40.82), husband/partner substance use (AOR = 4.51, 95% CI: 1.44–14.20), and ever heard about the side effects of substance use (AOR = 14.60, 95% CI: 5.31–17.65) were factors significantly associated with maternal substance use during pregnancy.

**Conclusion:**

Nearly one in four pregnant women reported using substances during pregnancy. Educational and counseling interventions during antenatal care should emphasize the risks of substance use on maternal health and fetal development, particularly targeting women with a history of substance use or unplanned pregnancies. Efforts should also involve partners and employ community-based strategies to raise awareness of these risks.

## Introduction

Substance use is the consumption of harmful stimulant substances, including alcohol, tobacco products, caffeine, khat leaves, illicit drugs, inhalants, and other substances that can be ingested, inhaled, injected, or absorbed into the body, potentially leading to dependence and adverse effects on physiological, mental, physical, or emotional functions (1, 2). Substance use is a major preventable contributor to the global burden of disease (3). Tobacco, alcohol, and other illicit drugs are recognized by the World Health Organization (WHO) as leading contributors to ill health worldwide (4).

Women are more vulnerable to substance use during their reproductive years, which means that those who are pregnant or planning to become pregnant face a higher risk of substance abuse (5, 6). Substance use during pregnancy is a major public health issue due to its harmful effects on both the mother and the fetus (6–8). This includes an increased risk of inadequate parenting, miscarriage, ectopic pregnancy, premature rupture of membranes, placental abruption, intrauterine growth restriction, early labor, spontaneous abortion, infant mortality, stillbirth, congenital anomalies, neonatal abstinence syndrome, Fetal Alcohol Spectrum Disorder (FASD), low birth weight, and preterm delivery (6, 7, 9–12).

According to the WHO, women comprise about 40% of the global substance-using population (13). The prevalence of substance use during pregnancy varies widely across countries, regions, and ethnic groups (14). Tobacco is the most commonly used substance during pregnancy, followed by alcohol, cannabis, and other illicit drugs (6). In Sub-Saharan Africa, substance use during pregnancy ranges from 2.2% to 36.5% (15–17), while in East African countries, including Ethiopia, it ranges from 11.3% to 60% (18–23). Previous studies have identified various factors associated with substance use during pregnancy, including maternal age, maternal and partner/husband's education level, monthly income, occupation, partner/husband's substance use, lack of antenatal care, gestational age, pregnancy status, pre-pregnancy substance use, and place of residence (6, 17, 19, 20, 23–25).

Globally, various strategies have been introduced to address substance use during pregnancy, including the WHO guidelines for identifying and managing substance use in expectant mothers (26, 27). In Ethiopia, efforts to limit cigarette smoking and khat chewing have been made, but enforcement varies across regions. Cultural practices also pose significant challenges. For instance, alcohol consumption is deeply embedded in social and religious traditions, particularly in rural areas, where alcoholic beverages are commonly consumed during social gatherings and ceremonies. Additionally, khat chewing is not only a social norm in many communities but also serves ritualistic and economic purposes, making its reduction particularly complex. These deeply rooted cultural norms hinder public health efforts to reduce substance use during pregnancy (20, 23).

Although limited studies have examined maternal substance use in Ethiopia, particularly during pregnancy, evidence remains scarce, especially within the study area. A thorough review of the literature indicates that while some study exists at the national level, there is a lack of location-specific data on the prevalence and associated factors of substance use among pregnant women in Adama, central Ethiopia. Given regional variations in substance use patterns and risk factors, localized data are crucial for designing effective interventions. Therefore, this study aimed to assess the prevalence and associated factors of maternal substance use in this setting. The findings will provide insights to guide targeted interventions, build awareness, provide effective care, improve maternal and child health outcomes, and contribute to the broader understanding of substance use during pregnancy and its implications for maternal and child well-being.

## Methods

### Study design and setting

A health facility-based cross-sectional study was conducted from February 1 to April 30, 2024, in Adama town, located in central Ethiopia. Adama is situated 99 km southeast of Addis Ababa, in the East Shoa zone of the Oromia region. According to the 2007 Census conducted by the Central Statistical Agency (CSA) of Ethiopia, the town has a total population of 220,212, comprising 108,872 men and 111,340 women (28). The town's healthcare system comprises one government hospital and eight health centers. The hospital, situated within the town, delivers preventive and curative services to a population exceeding five million. Meanwhile, the health centers collectively serve an estimated catchment population of nearly half a million.

### Study population and eligibility criteria

The source population consisted of all pregnant women attending antenatal care at public health facilities in Adama town. The study population included pregnant women attending antenatal care at selected public health facilities in Adama town during the study period. However, pregnant women who were seriously ill or unable to communicate were excluded from the study.

### Sample size determination and sampling procedure

The required sample size for this study was calculated using the single population proportion formula with the following assumptions: a 95% confidence interval (CI) (critical value Z*α*/2 = 1.96), a calculated margin of error of 4% (d = 0.04), and a proportion of substance use (*p* = 0.265) of 26.5%, based on a study conducted in Eastern Ethiopia (23). After accounting for a 5% non-response rate, the final sample size was calculated to be 490.

The public health facilities in the town were stratified into hospitals and health centers. Out of the eight health centers, four were selected through simple random sampling, while the hospital was chosen purposively. The total sample size was distributed proportionally among the selected facilities based on their average ANC patient flow over the preceding three months. Study participants were then selected independently from each facility using a systematic sampling technique. The sampling interval for each facility was determined by dividing the average number of pregnant women attending ANC in the previous three months by the required sample size for that facility.

## Variables of the study

### Dependent variable

The dependent variable was maternal substance use during pregnancy, defined as a self-reported exposure to at least one of the three substances (alcohol, khat, or tobacco) at any point during the current pregnancy before the interview, regardless of the dose or frequency (20, 23, 26).

Alcohol use was defined as the consumption of any alcoholic beverage at any time during the pregnancy, regardless of the amount or frequency. This included both industrially produced alcohol (containing ethanol or ethyl alcohol) and locally produced beverages such as Teji, Areke, or Tela (9, 20, 23).

Khat use was defined as chewing khat at any time during the pregnancy, regardless of the amount or frequency (20, 23, 29).

Tobacco use was defined as the use of any tobacco product at any time during the pregnancy. This included cigarettes, other smoking forms such as shisha/hookah and bidi, as well as non-smoking forms like snuff or chewing tobacco, regardless of the amount or frequency (20, 23, 30).

### Independent variables

The independent variables include socio-demographic and economic factors (age, religion, marital status, residence, educational status, occupational status, and monthly income), Obstetric and health-related characteristics [gravidity, parity, gestational age, pregnancy status, antenatal care (ANC) follow-up, history of adverse birth outcomes, and history of medical problems before or during pregnancy], and Behavioral-related characteristics (pre-pregnancy substance use, ever heard about the side effects of substance use, husband or partner substance use, and a history of intimate partner violence).

### Data collection tools and procedure

Data were collected using a structured, interviewer-administered questionnaire adapted from a review of previous literature (6, 19, 20, 22, 23, 25). The questionnaire included sections on sociodemographic and economic characteristics, obstetric and health-related factors, and behavioral characteristics relevant to mothers’ experiences during their pregnancy. Initially prepared in English, the questionnaire was translated into the local languages, Amharic and Afan Oromo, with back-translation performed to ensure accuracy.

Before the primary data collection, a pretest was conducted on 5% (*n* = 25) of the sample to assess clarity and reliability. Data collectors received two days of training on the data collection tools, procedures, and study objectives. Ten trained midwives collected the data. Mothers were interviewed in private places to ensure their privacy and to encourage participation. Regular meetings and continuous supervision were implemented to ensure quality and consistency throughout the data collection process.

### Data processing and statistical analysis

Data were entered using Epi-Info Version 7.2 and then exported to SPSS Version 25 for processing and analysis. Before analysis, the data were cleaned, coded, and categorized. Descriptive statistics were used to describe the characteristics of the participants, and the proportion of substance use was calculated with a 95% confidence interval. The association between independent variables and substance use was modeled using binary logistic regression. Initially, bivariable binary logistic regression analysis was conducted to identify variables with a crude association with substance use. At this level, variables with a *p*-value < 0.25 were considered candidates for inclusion in the multivariable logistic regression analysis. Subsequently, multivariable logistic regression analysis was performed to identify factors independently associated with substance use. The regression model was fitted using a standard model-building approach. Adjusted odds ratios (AOR) with 95% confidence intervals (CI) were used to estimate the strength of the association. The statistical significance of associations was declared for variables with a *p*-value < 0.05.

### Ethical consideration

Ethical approval was obtained from the Institutional Review Board of Adama General Hospital and Medical College. A permission letter was subsequently submitted to the Adama Town Health Office and public health facilities, where approval was granted to conduct the study. Participants were fully informed about the study's potential benefits and risks, and their voluntary participation or refusal was documented through written consent. Privacy and anonymity were rigorously maintained to protect participants’ rights and ensure confidentiality. All procedures adhered to the principles outlined. All procedures were conducted under the ethical principles outlined in the Helsinki Declaration.

## Result

### Socio-demographic and economic characteristics

A total of 472 study participants were included in this study, with a response rate of 96.3%. Nearly half 248 (52.5%) of the participants were aged 24 years or younger, and the majority 383 (81.1%) resided in urban areas. In terms of religion, 199 participants (42.2%) were Orthodox Christians. Most participants 433 (91.7%) were married, while 152 (32.2%) had completed elementary education, and 315 (66.7%) were housewives (Table 1).

**Table 1:** Socio-demographic and economic characteristics of pregnant women in Adama, central Ethiopia (*n* = 472).

Variables	Categories	Frequency	Percentage
Age	≤ 24	248	52.5
	25–34	148	31.4
	≥ 35	76	16.1
Place of residence	Urban	383	81.1
	Rural	89	18.9
Religion	Orthodox	199	42.2
	Muslim	158	33.5
	Protestant	104	22.0
	Others*	11	2.3
Marital status	Married	433	91.7
	Unmarried	39	8.3
Women educational status	No formal education	42	8.9
	Elementary	152	32.2
	Secondary	180	38.1
	College and above	98	20.8
Husband/partner educational status	No formal education	39	8.3
	Elementary	102	21.6
	Secondary	189	40.0
	College and above	142	30.1
Women occupation	Housewife	315	66.7
	Farmer	13	2.8
	Merchant	74	15.7
	Government employee	63	13.3
	Others**	7	1.5
Husband/partner occupation	Farmer	36	7.6
	Merchant	176	37.3
	Government employee	131	27.8
	Private employee	98	20.8
	Others***	31	6.5
Total family size	< 5	255	54.0
	≥ 5	217	46.0
Average monthly income (ETB)	< 1,500	21	4.4
	1,500–3,499	78	16.5
	3,500–4,999	195	41.3
	≥ 5,000	178	37.7

* Catholic, Wakefeta.

** Student, housemaid, waitress.

*** Non-governmental employee, daily laborer, driver, unemployed/jobless.

### Obstetrics and health-related characteristics

In this study, 341 participants (72.2%) were multigravida, and 281 (59.5%) were multiparous. The majority, 281 (80.7%), had attended antenatal care (ANC) follow-ups. Additionally, 203 participants (43%) were in their second trimester of pregnancy, and 392 pregnancies (83.1%) were planned (Table 2).

**Table 2:** Obstetrics and health-related characteristics of pregnant women in Adama, central Ethiopia (*n* = 472).

Variables	Categories	Frequency	Percentage
Gravidity	Primigravida	131	27.8
	Multigravida	341	72.2
Parity	Nullipara	132	28.0
	Primipara	59	12.5
	Multi para	281	59.5
ANC follow-up	Yes	381	80.7
	No	91	19.3
Number of ANC visits (*n* = 381)	1–3	329	86.4
	≥ 4	52	13.6
Gestational age	First trimester	95	20.1
	Second trimester	203	43.0
	Third trimester	174	36.9
Pregnancy status	Planned	392	83.1
	Unplanned	80	16.9
History of adverse birth outcome (*N* = 341)	Yes	53	15.5
	No	288	84.5

ANC, antenatal care.

### Maternal substance use and behavioral-related characteristics

In this study, the overall prevalence of substance use during pregnancy was 22.0% (95% CI: 18.2–26.0). Among the substances used during the current pregnancy, alcohol was the most common (97.1%), followed by khat (51.0%) and tobacco (33.7%). Moreover, 177 participants (47.5%) reported a history of substance use before the current pregnancy, while 296 (62.7%) stated that their husband or partner used substances (Table 3).

**Table 3:** Maternal substance use and other behavioral-related characteristics of pregnant women in Adama, central Ethiopia (*n* = 472).

Variables	Categories	Frequency	Percentage
Substance use during the current pregnancy	Yes	104	22.0
	No	368	78.0
Type of substance used (*n* = 104) (*multiple answers possible*)	Alcohol	101	97.1
	Khat	53	51.0
	Tobacco	35	33.7
Reason for using the substance (*n* = 104) (*multiple answers possible*)	For relaxation	56	53.8
	Socialization	43	41.3
	Peer pressure	21	20.2
	Others*	7	6.7
Pre-pregnancy substance use	Yes	177	37.5
	No	295	62.5
Type of substance used (*n* = 177) (*multiple answers possible*)	Alcohol	99	55.9
	Khat	83	46.9
	Tobacco	36	20.3
Reason for using the substance (*n* = 177) (*multiple answers possible*)	For relaxation	82	46.3
	Socialization	78	44.1
	Peer pressure	34	19.2
	Others**	13	7.3
Ever been advised to stop using substances (*n* = 177)	Yes	25	14.1
	No	152	85.9
Husband/partner substance use	Yes	296	62.7
	No	176	37.3
Type of substance used (*n* = 296) (*multiple answers possible*)	Alcohol	123	41.6
	Khat	110	37.2
	Tobacco	85	28.7
Ever heard about the side effects of substance use	Yes	176	37.3
	No	296	62.7
History of intimate partner violence	Yes	155	32.8
	No	317	67.2

* Medicinal purposes, religious perspectives, cultural reasons.

** Unaware of harm, cultural reasons.

### Factors associated with substance use

In the multivariable binary logistic regression analysis, pregnancy status, pre-pregnancy substance use, husband/partner substance use, and awareness of the side effects of substance use were identified as statistically significant factors associated with substance use during pregnancy.

In this study, women with unplanned pregnancies had 3.5 times greater odds of substance use during pregnancy compared to those with planned pregnancies (AOR = 3.49, 95% CI: 1.23–9.89). The odds of substance use during pregnancy was higher among women with a history of pre-pregnancy substance use compared to their counterparts (AOR = 24.16, 95% CI: 11.49–40.82). This study also revealed that pregnant women with substance-user husbands/partners had 4.5 times higher odds of substance use during pregnancy compared to women with non-user husbands (AOR = 4.51, 95% CI: 1.44–14.20). Furthermore, women who had never heard about the side effects about the side effects of substance use had higher odds of substance use during pregnancy compared to those who were aware of the side effects (AOR = 14.60, 95% CI: 5.31–17.65) (Table 4).

**Table 4:** Factors associated with maternal substance use during pregnancy pregnant women in Adama, central Ethiopia (*n* = 472).

Variables	Categories	Substance use		COR (95% CI)	AOR (95% CI)
		No	Yes		
Parity	Nullipara	92	40	1	1
	Primipara	43	16	0.86 (0.47–1.86)	2.59 (0.80–8.38)
	Multi para	233	48	0.47 (0.44–0.89)*	1.35 (0.60–3.02)
Gestational age	First trimester	70	25	1	1
	Second trimester	151	52	0.96 (0.55–1.68)	0.49 (0.20–1.18)
	Third trimester	147	27	0.51 (0.28–0.95)*	0.31 (0.12–1.17)
Pregnancy status	Unplanned	160	95	6.19 (4.71–8.13)*	3.49 (1.23–9.89)**
	Planned	198	19	1	1
Pre-pregnancy substance use	Yes	87	90	20.76 (11.26–38.30)*	24.16 (11.49–40.82)**
	No	281	14	1	1
Husband/partner substance use	Yes	200	96	10.08 (4.76–21.34)*	4.51 (1.44 −14.20)**
	No	168	8	1	1
Ever heard about the side effects of substance use	Yes	169	7	1	1
	No	199	97	11.77 (5.32–26.03)*	14.60 (5.31–17.65)**
History of intimate partner violence	Yes	104	51	2.44 (1.56–3.82)*	1.90 (0.91–3.96)
	No	264	53	1	1

AOR, adjusted odds ratio; CI, confidence interval; COR, crude odds ratio.

* Significant at *p*-value <0.25 in unadjusted logistic regression analysis.

** Significant at *p* < 0.05 in adjusted logistic regression analysis, 1 = Reference.

## Discussion

Substance abuse and dependency during pregnancy remain a pressing issue in many developing countries, including Ethiopia. This study assessed the prevalence and factors associated with maternal substance use during pregnancy in Adama, Central Ethiopia.

The prevalence of substance use among pregnant women in this study was 22.0% (95% CI: 18.2–26.0), which is comparable to a study conducted in the United States (25.8%) (31). However, the prevalence found in this study is lower than that reported in studies from Northeast Ethiopia (48.1%) (20), Eastern Ethiopia (26.5%) (23), South Central Ethiopia (60.1%) (22), Amhara, Ethiopia (38.3%) (25), and Jimma, Ethiopia (37.9%) (19). In contrast, the prevalence of substance use in this study is higher than that reported in a study from Southeast Iran (15%) (32) and the United States (33). The observed differences might be attributed to variations in cultural attitudes toward substance use, social norms, and the distribution and availability of substances. Additionally, differences in how substance use is defined and measured, as well as variations in study methodologies, sample sizes, and population characteristics, could contribute to the discrepancies in reported prevalence.

This study found that pregnant women with unplanned pregnancies had greater odds of substance use compared to those with planned pregnancies. This finding is consistent with studies conducted in Iran (32) and Northeast Ethiopia (20). A possible explanation for this is that women with unplanned pregnancies may be less likely to seek prenatal care or counseling from healthcare providers or family members. As a result, they may be more vulnerable to engaging in harmful behaviors, including the use of substances. Moreover, unplanned pregnancies often lead to emotional distress, and women may turn to substances like alcohol or drugs as a coping mechanism.

In this study, the odds of substance use during pregnancy was higher among women with a history of pre-pregnancy substance use compared to their counterparts. This finding is similar to previous studies (23, 34). This can be explained by the continuity of behaviors and the challenges associated with breaking established habits. Women who use substances before pregnancy may face difficulties in stopping due to addiction or dependency, and their established patterns of use may persist into the prenatal period.

Consistent with previous studies conducted in Iran (32) and Ethiopia (19, 20, 23), this study found that pregnant women with substance-using husbands/partners had higher odds of substance use during pregnancy compared to women with non-substance-using husbands/partners. This may be attributed to the influence of their partners’ behaviors. Substance use is often a shared activity in relationships, and women may be more likely to engage in substance use themselves if their partners also use substances. Additionally, the normalization of substance use within the household can reduce perceived risks and increase the likelihood of similar behaviors during pregnancy.

Moreover, the current study indicated that women who had never heard about the side effects of substance use had higher odds of substance use during pregnancy compared to those who were aware of the side effects. The possible justification could be that women who lack awareness of the side effects of substance use during pregnancy are more likely to engage in such behaviors due to a limited understanding of the potential risks to maternal and fetal health. Knowledge serves as a critical deterrent, as awareness of the harmful effects can motivate pregnant women to avoid substances. Without this information, they may not perceive substance use as harmful, increasing their likelihood of use during pregnancy (35, 36).

This study has some limitations. It relied on self-reported information from mothers, which introduces the possibility of recall and reporting bias. Moreover, social desirability bias may have influenced participants to respond in ways they deemed socially acceptable. The inclusion of pregnant women at various gestational ages may affect the consistency of substance use patterns throughout pregnancy. Lastly, as a cross-sectional study, it shares the inherent limitations associated with this study design.

## Conclusion

This study revealed that approximately one-fifth of pregnant women reported using substances (alcohol, khat, or cigarettes) during pregnancy. Factors significantly associated with substance use during pregnancy included unplanned pregnancy, pre-pregnancy substance use, substance use by the husband/partner, and lack of awareness of its side effects. Educational and counseling interventions during antenatal care should emphasize the risks of substance use on maternal health and fetal development, particularly targeting women with a history of substance use or unplanned pregnancies. It is also important to involve husbands and partners in these efforts, as their behavior can influence the use of substances. Furthermore, community-based strategies are needed to increase overall awareness of the risks associated with substance use during pregnancy.

## Data Availability

The original contributions presented in the study are included in the article/Supplementary Material, further inquiries can be directed to the corresponding author.
